# Neuronal hypoxia *in vitro*: Investigation of therapeutic principles of HUCB-MNC and CD133^+ ^stem cells

**DOI:** 10.1186/1471-2202-9-91

**Published:** 2008-09-19

**Authors:** Doreen M Reich, Susann Hau, Tobias Stahl, Markus Scholz, Wilfried Naumann, Frank Emmrich, Johannes Boltze, Manja Kamprad

**Affiliations:** 1 Fraunhofer-Institute for Cell Therapy and Immunology, Perlickstraße 1, 04103 Leipzig, Germany; 2 University of Leipzig, Faculty of Veterinary Medicine, Department of Anatomy, Histology and Embryology, An den Tierkliniken 43, 04103 Leipzig, Germany; 3 University of Leipzig, Institute of Medical Informatics, Statistics and Epidemiology, Haertelstrasse 16-18, 04107 Leipzig, Germany; 4 University of Leipzig, Faculty of Biosciences, Pharmacy and Psychology, Institute of Biology II, Talstrasse 33, 04103 Leipzig, Germany; 5 Translational Centre for Regenerative Medicine, Philipp-Rosenthal-Strasse 55, 04103 Leipzig, Germany; 6 University of Leipzig, Institute of Clinical Immunology and Transfusion Medicine, Johannisallee 30, 04103 Leipzig, Germany

## Abstract

**Background:**

The therapeutic capacity of human umbilical cord blood mononuclear cells (HUCB-MNC) and stem cells derived thereof is documented in animal models of focal cerebral ischemia, while mechanisms behind the reduction of lesion size and the observed improvement of behavioral skills still remain poorly understood.

**Methods:**

A human *in vitro *model of neuronal hypoxia was used to address the impact of total HUCB-MNC (tMNC), a stem cell enriched fraction (CD133^+^, 97.38% CD133-positive cells) and a stem cell depleted fraction (CD133^-^, 0.06% CD133-positive cells) of HUCB-MNC by either direct or indirect co-cultivation with post-hypoxic neuronal cells (differentiated SH-SY5Y). Over three days, development of apoptosis and necrosis of neuronal cells, chemotaxis of MNC and production of chemokines (CCL2, CCL3, CCL5, CXCL8, CXCL9) and growth factors (G-CSF, GM-CSF, VEGF, bFGF) were analyzed using fluorescence microscopy, FACS and cytometric bead array.

**Results:**

tMNC, CD133^+ ^and surprisingly CD133^- ^reduced neuronal apoptosis in direct co-cultivations significantly to levels in the range of normoxic controls (7% ± 3%). Untreated post-hypoxic control cultures showed apoptosis rates of 85% ± 11%. tMNC actively migrated towards injured neuronal cells. Both co-cultivation types using tMNC or CD133^- ^reduced apoptosis comparably. CD133^- ^produced high concentrations of CCL3 and neuroprotective G-CSF within indirect co-cultures. Soluble factors produced by CD133^+ ^cells were not detectable in direct co-cultures.

**Conclusion:**

Our data show that heterogeneous tMNC and even CD133-depleted fractions have the capability not only to reduce apoptosis in neuronal cells but also to trigger the retaining of neuronal phenotypes.

## Background

Transplantation of adult stem cells has been shown to be an auspicious and effective treatment for degenerative and traumatic neurological diseases [1]. Among degenerative neurological disorders acute ischemic stroke is the leading cause of disability and death in industrial nations [2-4].

Acute stroke leads to an increased release of hematopoietic stem and progenitor cells from bone marrow into peripheral blood [5]. It is assumed that these cells take part in self-healing processes occurring after neuronal injury. They are supposed to promote the survival of the injured brain tissue by producing neurotrophic factors [6], to enhance endogenous angiogenesis [7] and neurogenesis [8] or even to transdifferentiate into neuronal cells [9]. However, the stroke induced endogenous release of hematopoietic stem and progenitor cells seems not to be sufficient to compensate massive loss of brain tissue after extended ischemic stroke. Therefore, external application of hematopoietic stem and progenitor cells is expected to complement current treatment of acute stroke based on thrombolytic therapy. An appropriate source of hematopoietic stem cells is the mononuclear cell (MNC) fraction of human umbilical cord blood (HUCB) [10-12]. Transplantation of HUCB-MNC as well as enriched HUCB hematopoietic stem cells into animals which were subjected to focal stroke caused by middle cerebral artery occlusion (MCAO) ameliorated the animals' functional outcome and reduced the lesion size [13]. However, there are still manifold unanswered questions addressing the beneficial influence of such grafts on injured neuronal cells.

It has been documented that there is no neuronal transdifferentiation of hematopoietic stem cells in vitro [14-16]. Though so far there is no convincing proof that locally administered hematopoietic stem cells transdifferentiate into functionally neuronal cells forming the basis of the animals' behavioral progression [17].

It has recently been shown that there is no need for MNC to enter the brain for neuroprotection. Soluble factors like GDNF, NGF, BDNF or G-CSF are known to promote neuroprotection over long-distances [18,19]. This raises many questions about the cellular mechanisms causing the functional improvement after grafting [20]. Prevention of neurons from apoptotic cell death [21] is considered to be supported by the transplantation and could be directly connected to improved tissue conservation, lesion size reduction and superior functional outcome [22].

Cell culture models of neuronal hypoxia complement the exploration of particular interactions between grafts and neuronal tissue. Our study is based on a well established post-hypoxic neuronal cell culture model (SH-SY5Y). This model was used to address (i) the neuroprotective potential of stem cell enriched and -depleted HUCB derived cell fractions, (ii) the impact of these cells especially on apoptotic status of oxygen-deprived neurons, and (iii) the mediation of cell-derived survival signals (soluble or cell-attached).

## Results

### Direct co-cultivation with each fraction of HUCB-MNC reduced apoptosis in post-hypoxic neuronal cells

**Figure 1:**
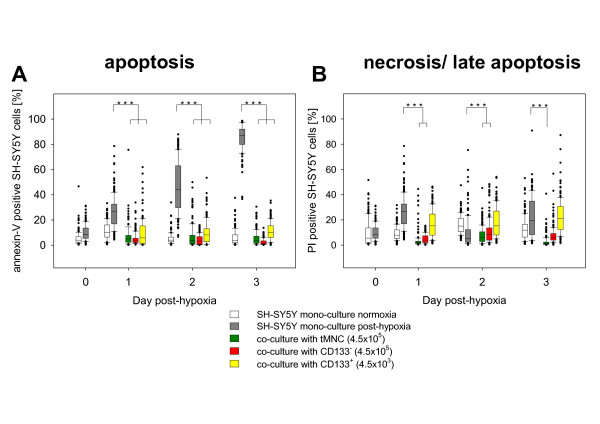
**Time course of apoptosis and necrosis of post-hypoxic neuronal cells in direct co-cultures with tMNC, CD133^+ ^and CD133^-^**. Box plots showing time course of apoptosis (A) and necrosis/late apoptosis (B) in direct co-cultures of post-hypoxic neuronal cells with tMNC, CD133^- ^or CD133^+ ^(colored plots). Grey boxes display the development of cell death in post-hypoxic neuronal cells directly after hypoxic pre-cultivation of SH-SY5Y cells (Day 0) and 1–3 days post-hypoxia. The ordinate represents the percentage of apoptotic and necrotic cells, respectively. Rates of apoptosis and necrosis were calculated in relation to the total number of SH-SY5Y cells.

Hypoxic cultivation (48 hours) of fully matured neuronal SH-SY5Y cells resulted in an initial rate of apoptosis of 26% ± 13%. Within the following three days rate of apoptosis increased to 85% ± 11%. By contrast, normoxic control cultures showed a stable amount of apoptotic cells (7% ± 3%) over the whole observation time (data not shown). Direct co-cultivation with tMNC and CD133^- ^showed pronounced reduction of neuronal apoptosis. Similar results were obtained after application of CD133^+^. By application of 4.5 × 10^3 ^CD133^+ ^they were given in equal amounts as they exist in tMNC. Though the whole cell amount of CD133^+ ^was 100 times less then tMNC administered (Fig. 1A).

Levels of necrosis in post-hypoxic control cultures remained nearly stable (approximately 25%) over three days. tMNC and CD133^- ^cell application also induced a significant reduction of necrosis. CD133^+ ^cells did not influence the level of necrosis (Fig. 1B).

### In indirect co-cultures tMNC and CD133^- ^were also sufficient to decrease apoptosis of post-hypoxic neuronal cells

**Figure 2:**
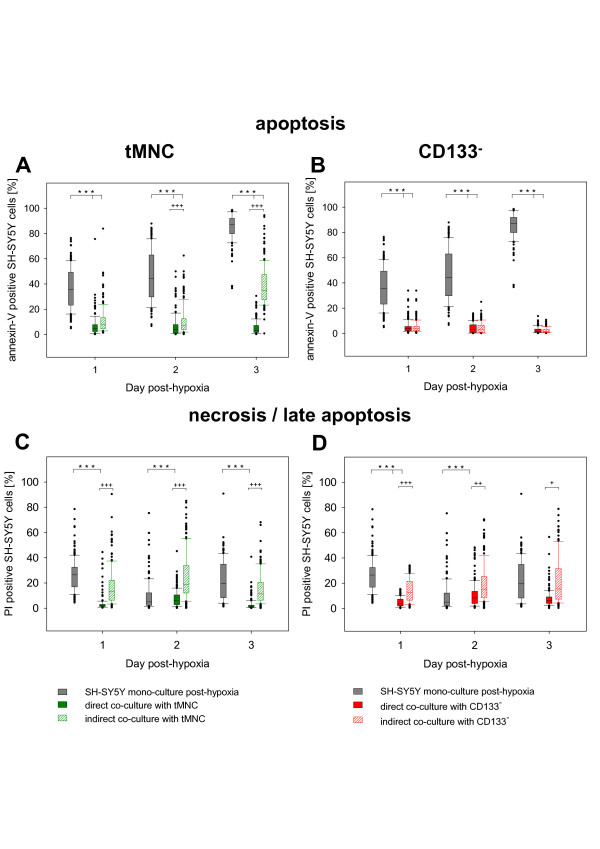
**Effects of direct and indirect application of tMNC and CD133^- ^on apoptosis and necrosis of post-hypoxic neuronal cells**. Box plots represent the percentage of annexin-V positive post-hypoxic neuronal cells (A, B) and of PI positive post-hypoxic neuronal cells (C, D) in direct and indirect co-cultures. Co-cultures were performed with tMNC (A, C) and CD133^- ^(B, D). The extents of apoptosis (annexin-V-binding) and necrosis (PI) were analyzed for three days after hypoxia.

Over the entire observation period, direct as well as indirect co-cultivation with tMNC or CD133^- ^exhibited a significant reduction of apoptosis. In all co-culture set-ups percentage of annexin-V positive cells was significantly lower (p ≤ 0.001) than in post-hypoxic control cultures (Fig. 2A, B). Direct and indirect co-cultivation of CD133^-^resulted in similar rates of apoptosis continuously below 5% of annexin-V positive cells (Fig. 2B). However, still generating strong neuroprotective effects, the number of apoptotic neuronal cells in indirect tMNC co-cultures was significantly higher than in direct co-cultures at Day 2 and Day 3 (p ≤ 0.001) as shown in figure 2A. Direct co-cultivation with tMNC resulted in a stable level of 6% ± 1% neuronal apoptosis and was therefore significantly lower than in post-hypoxic control cultures (Day 2: 46% ± 20%; Day 3: 85% ± 11%).

When tMNC were indirectly co-cultured neuroprotection was as pronounced as in direct co-cultures on the first day after hypoxia. Two and three days after application there was still a significant, but compared to control cultures reduced, protective effect in the indirect co-cultures while protection in direct co-cultures was as distinct as on Day 1 (7% ± 8%).

The comparison of both application types using tMNC and CD133^- ^showed that soluble factors seem to have strong therapeutic potential (Fig. 2A).

**Figure 3:**
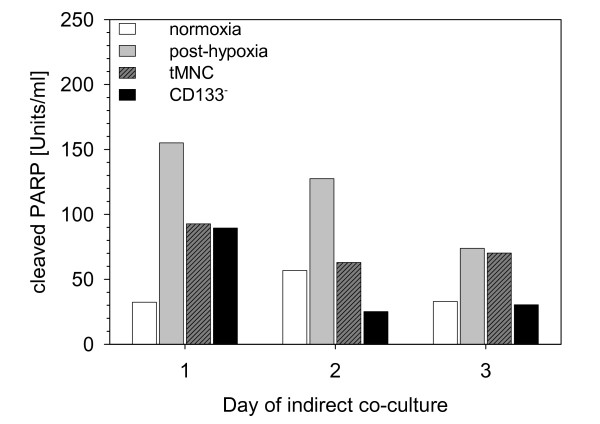
**Direct comparison of effects of tMNC and CD133^- ^on late apoptosis (PARP cleavage)**. Bar chart showing concentrations of cleaved PARP in lysates of post-hypoxic neuronal cells which were indirectly co-cultivated with tMNC (grey shaded bars) or CD133^- ^(black bars). Controls are post-hypoxic mono-cultures (grey bars) and normoxic cultures (white bars). Each bar visualizes pooled samples obtained from three independent experiments.

The positive influence of indirect co-cultivation on the amount of apoptotic cells, revealed by annexin-V detection, was also confirmed by typical patterns in the cleavage of PARP, a late marker of apoptosis (Fig. 3). In indirect co-cultures with tMNC and CD133^- ^quantities of cleaved PARP were nearly at the same level on Day 1 post-hypoxia. On Day 2 and Day 3 neuroprotection by CD133^- ^resulted in concentrations of cleaved PARP ranging in the level of the normoxic control (Fig. 3).

Only direct co-cultures of tMNC and CD133^- ^displayed improved protection from necrosis/late apoptosis as revealed by Propidium Iodide labeling of post-hypoxic neuronal cells (Fig. 2C, D). Indirect co-cultivation did not reduce the percentage of necrotic/late apoptotic neuronal cells.

### tMNC localized in close proximity to post-hypoxic neuronal cells

**Figure 4:**
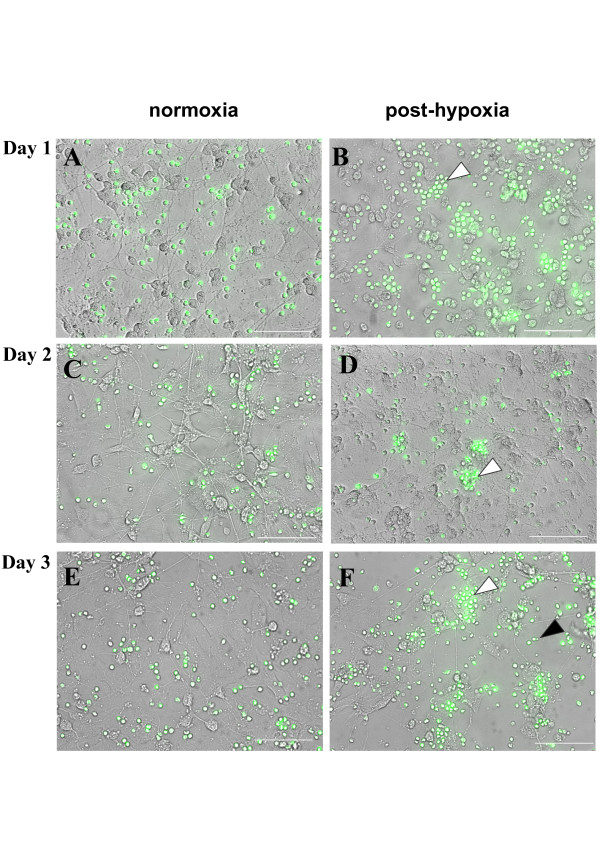
**Distribution of tMNC in direct co-cultures**. Phase contrast fluorescence micrographs of co-cultures of neuronal cells (unstained) and tMNC (green). Representative images show post-hypoxic neuronal cultures (B, D, F) in contrast to normoxic control cultures (A, C, E) over a time course of three days. Images illustrate the distribution of tMNC in relation to neuronal cells. White arrows point at spatial accumulation of tMNC which are clustered around neuronal somata and processes in post-hypoxic cultures (B, D, F). In normoxic cultures no clustering of tMNC was observed (A, C, E). The black arrow points at a conserved neuronal morphology including branched processes. Scale bars indicate 100 μm.

In direct co-cultures many tMNC were found in close spatial relation with post-hypoxic neuronal cells already at Day 1 (Fig. 4B). This became even more evident at later time points (Fig. 4D, F). At Day 3, the vast majority of tMNC was found adjacent to neuronal somata and processes (Fig. 4F). Interestingly, co-cultivation with tMNC seemed to have strong positive effects on the preservation of typical neuronal cell morphology as the formation of branched processes.

In contrast, in normoxic control cultures tMNC were evenly spread throughout the culture dish (Fig. 4A, C, E).

### Cytokine secretion patterns induced by direct co-cultivation with tMNC or CD133^- ^were similar; cytokines produced by CD133^+ ^were not detectable

**Figure 5:**
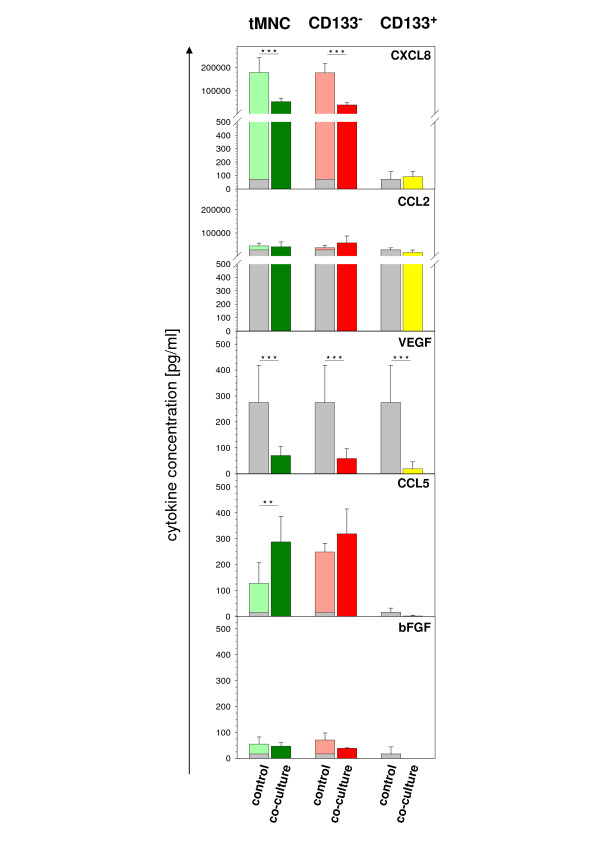
**Cytokine profile in direct co-cultures with tMNC, CD133^+^and CD133^- ^on Day 3**. **Control: **sum of cytokine concentration from single cultured (grey) post-hypoxic SH-SY5Y cells and HUCB-MNC (light green = tMNC, light red = CD133^-^, not detectable = CD133^+^). **Co-culture**: direct co-culture of post-hypoxic SH-SY5Y cells and HUCB-MNC (dark green = tMNC, dark red = CD133^-^, dark yellow = CD133^+^). Data are derived from four independent experiments and are expressed as pg/ml. * significant differences in cytokine concentrations of co-cultures compared to control cultures. Note different axis scaling.

In supernatants of direct co-cultures and corresponding mono-cultures we assessed concentrations of CCL2, CCL5, CXCL8, VEGF and bFGF (Fig. 5).

Post-hypoxic neuronal cells secreted the chemokines CXCL8 and CCL2 and the growth factor VEGF. Low amounts of CCL5 and bFGF were detectable, as well. Mono-cultures of tMNC and CD133^- ^displayed similar growth factor and chemokine secretion patterns: CXCL8 and CCL2 were produced in considerable amounts while CCL5 and bFGF were secreted at low levels. VEGF was not present. None of the investigated soluble factors was detected in supernatants of purified CD133^+ ^mono-cultures.

In direct co-cultures with tMNC or CD133^- ^secretion of cytokines was regulated very similar: reduction of CXCL8 (p ≤ 0.001) and VEGF (p ≤ 0.001) and increase of CCL5 (Fig. 5). CD133^+ ^co-cultivation only influenced VEGF levels, which decreased significantly (Fig. 5).

### Up-regulation of soluble factors in indirect CD133^- ^co-cultures was associated with long term neuroprotection

In a second set of experiments we investigated different effects of direct and indirect co-culturing on cytokine secretion. Therefore, concentrations of CXCL9, CCL3, VEGF, G-CSF and GM-CSF were measured in normoxic and post-hypoxic mono-cultures, and co-cultures with tMNC and CD133^-^.

**Figure 6:**
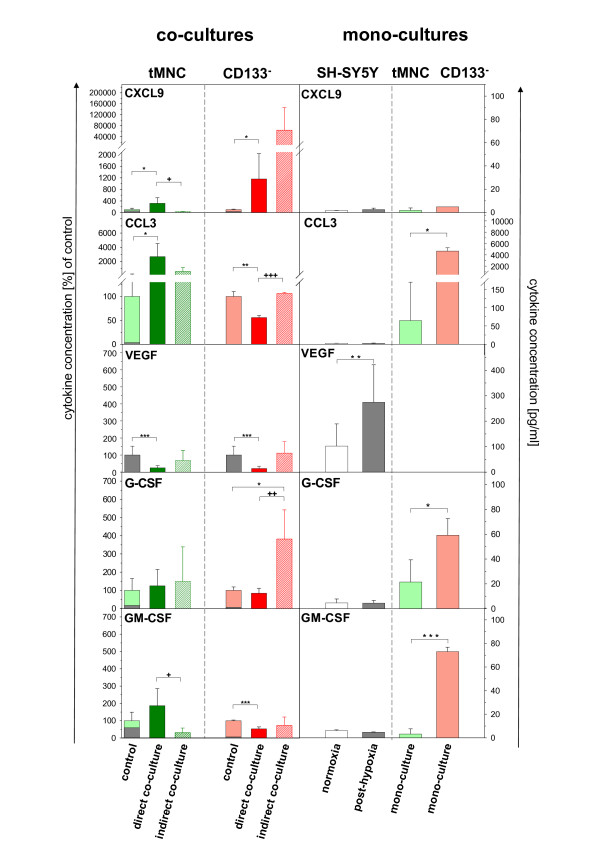
**Effect of co-cultivation system (direct v. indirect) on cytokine secretion during co-cultivation of post-hypoxic neuronal cells with tMNC and CD133^-^**. **Control**: sum of cytokine concentration from single cultured (grey) post-hypoxic SH-SY5Y cells and HUCB-MNC (light green = tMNC, light red = CD133^-^). **Co-cultures**: co-culture of post-hypoxic SH-SY5Y cells and tMNC (direct: dark green, filled; indirect: dark green, striped), co-culture of post-hypoxic SH-SY5Y cells and CD133^- ^(direct: dark red, filled; indirect: dark red, striped). Effects are displayed in percent as relative changes to control. **Mono-cultures**: absolute cytokine concentrations in supernatants of SH-SY5Y normoxic and post-hypoxic mono-cultures (white or grey bars, respectively) and tMNC (light green bars) or CD133^- ^(light red bars). * significant changes to control. + significant differences between co-cultivation systems.

Except VEGF neuronal cells did not secret detectable amounts of either CXCL9, CCL3, G-CSF nor GM-CSF in mono-cultures. Both, tMNC and CD133^- ^cells expressed CCL3 and G-CSF but only CD133^- ^produced GM-CSF (Fig. 6).

In co-cultures with tMNC direct co-cultivation generated more pronounced effects than indirect co-cultivation, as seen for the relative up-regulation of CXCL9, GM-CSF (both, p ≤ 0.05) and CCL3 and the relative down-regulation of VEGF.

Only direct CD133^- ^co-cultivation had an impact on regulation of CCL3, VEGF and GM-CSF. Secretion of all three cytokines was markedly decreased compared to controls whereas indirect co-cultivation with CD133^- ^had no effect. In contrast, the secretion of CXCL9 and G-CSF (p ≤ 0.01) was markedly induced by indirect co-cultivation with CD133^-^. For these cytokines direct co-cultivation showed no effect (G-CSF) or resulted in a remarkably reduced production (CXCL9) as compared to indirect co-cultivation with CD133^- ^(Fig. 6).

The experiment also revealed the influence of different HUCB-MNC-derived cell preparations on the cytokine secretion.

Most prominent, direct co-cultivation with tMNC significantly increased expression of CCL3 (p ≤ 0.05) whereas direct co-cultivation with CD133^- ^resulted in a relative reduction of this chemokine (p ≤ 0.01).

## Discussion

It has been previously shown that HUCB derived MNC as well as nearly pure stem cell populations obtained from MNC are able to improve the clinical outcome of animals after MCAO [23]. In an *in vitro *model of neuronal hypoxia we discern cell populations within MNC being able to improve neuronal survival and disclose potential neuroprotective mechanisms.

First we studied the effects of tMNC application on the apoptotic status of post-hypoxic neurons. We found that direct application of tMNC results in preservation of neuronal morphology based on a constant protection from apoptosis (Fig. 2A).

For *in vivo *experiments it has been described that none or only a minority of systemically administrated cells were detected in the brain while large quantities were found in the spleen, in the lungs and in the blood of the animals. Nevertheless, some studies showed that cell-treatment improved behavioral deficits [18,23,24]. This indicates the importance of soluble factors for neuroprotection. Our data obtained from indirect co-cultures strongly support this hypothesis. tMNC application was highly protective, although the anti-apoptotic effect was slightly weakened after two days (Fig. 2A). Probably, soluble factors act as "first aid" messengers while longer protection seems to demand close proximity between tMNC and neuronal cells. The comparison of direct application of tMNC to normoxic and post-hypoxic neuronal cells revealed that only post-hypoxic neuronal cells attracted tMNC (Fig. 4). The clustering of tMNC around neuronal cells could explain the enhanced protection from apoptosis in direct co-cultures with tMNC at Day 2 and Day 3 (Fig. 4, Fig. 2A). Spatial contiguity is possibly associated with higher concentrations of neuroprotective mediators in the micro-environment of injured neuronal cells. Post-hypoxic neuronal cells were shown to significantly up-regulate the adhesion molecule ICAM-1 following hypoxia [25]. Hence, ICAM-1 (CD54) expression could underlay the observed co-localization via binding of LFA-1 (CD11) [26]. Besides adhesion molecules, chemotaxis could mediate this cellular co-localization. VEGF, known to exert chemotactic effects on monocytes [27], was produced by post-hypoxic SH-SY5Y cells (Fig. 6).

To focus the neuroprotective effects of stem cells we enriched or depleted CD133^+ ^cells from HUCB-MNC. Purities of 97.38% for the stem cell preparation and a reduction of CD133^+ ^up to 0.06% for the stem cell depleted fraction were achieved. In direct co-cultures CD133^+ ^were applied to neuronal cells in quantities of 4.5 × 10^3 ^resembling 1% of the applied tMNC, since CD133^+ ^account for about 1% of total cell number in tMNC preparations. Neuroprotective capacity of nearly pure CD133^+ ^cells was comparable to tMNC (Fig. 1A). According to our analyses of cytokine production CD133^+ ^cells did not secrete measureable cytokines neither in mono-cultures nor in co-cultures with injured neuronal cells (Fig. 5). Additional experiments using even tenfold elevated numbers of stem cells (4.5 × 10^4^) also revealed no detectable cytokine concentrations and did not result in an increased neuroprotective capacity (data not shown). Neuroprotection by the absence of measurable soluble mediators argues for a stem cell specific therapeutic mechanism through contiguity. Due to this assumption we did not include CD133^+ ^in the investigation of indirect co-cultures in this study.

Surprisingly, CD133^- ^cell fractions were also highly sufficient in protection from apoptosis. Therefore, the observed anti-apoptotic neuroprotective effects of tMNC in our experiments do not only relay on hematopoietic stem cell-specific mechanisms. This assumption is supported by the missing significance in the effect of CD133^+ ^on necrotic/late apoptotic loss of post-hypoxic neuronal cells (Fig. 1B). tMNC and CD133^- ^significantly reduced the percentage of PI-positive neuronal cells, whereas CD133^+ ^did not. Since CD133^- ^in indirect co-cultures were superior to tMNC in protection from apoptosis and because of very low amounts of CD133^+ ^in this preparation, CD133^- ^seem to mediate additional neuroprotective effects. Possibly, the separation process influenced the functionality of CD133^- ^cells. FACS analyses of the activation markers CD25, CD38, CD71 and HLA-DR on tMNC and on the separated CD133^- ^fraction did not reveal an increased population of activated cells in our preparations (data not shown). However, the expression of other activation molecules cannot be excluded, since this cell population displayed an enhanced secretion of cytokines like G-CSF, GM-CSF and CCL-3 (Fig. 6). We also cannot rule out that remaining CD34^+ ^cells could account for the neuroprotective activity of the CD133^- ^fraction, since depletion did only reduce the number of CD34^+ ^cells to 77% (Tab. 1).

**Table 1:** Cellular subfractions of MNC

**Cellular fractions of**	**Markers**	**Content in tMNC (%)**	**Content in CD133^- ^(%)**
I) Myeloid cells	CD14^+^/CD45^+^	14.1 ± 4.4	19.5 ± 2.4
II) Lymphocytes (including stem cells)		85.9 ± 4.4	80.5 ± 2.4

Subpopulations of II			
B-lymphocytes	CD19^+^/CD45^+^	12.3 ± 5.5	14.6 ± 0.5
T-lymphocytes	CD3^+^/CD45^+^	73.0 ± 10.2	76.8 ± 1.9
NK-cells	CD56^+^/CD45^+^	12.7 ± 4.5	8.3 ± 1.4
Hematopoietic stem/progenitor cells	CD133^+^/CD34^+^	1.2 ± 1.0	0.03 ± 0.01
	CD133^+^/CD34^-^	0.4 ± 0.2	0.03 ± 0.02
	CD133^-^/CD34^+^	0.4 ± 0.2	0.34 ± 0.06

Cellular fractions of HUCB-MNC after thawing of cryopreservated cells.

Subpopulations of lymphocytes and stem/progenitor cells are expressed as percent of total lymphocytes.

The investigation of soluble mediators exhibited that hypoxia induced a significant increase of VEGF in neuronal cells (Fig. 6, [28]). VEGF is documented to inhibit pro-apoptotic signaling by Bad (BCL2 antagonist of cell death), and cleavage of caspase-3, and caspase-9 [29] and therefore can be claimed as an autocrine self-protection mechanism of damaged neuronal cells. The neuroprotective impact of tMNC and CD133^-^cells in direct applications was accompanied by prevention of VEGF production which is typically induced in post-hypoxic neuronal cells (Fig. 5).

Neuroprotective effects of cell application could be mediated by G-CSF that was found only in mono-cultures of CD133^- ^but not in mono-cultures of post-hypoxic SH-SY5Y cells. Schneider et al., 2005 [30] pointed out that human SH-SY5Y neuroblastoma cells express the G-CSF receptor and that activation by the neurotrophic G-CSF reduced NO-induced poly-ADP ribose polymerase (PARP) and caspase-3 cleavage. Our data support these observations. At Day 3 in indirect co-cultures supply of G-CSF by CD133^- ^was associated with cleaved PARP levels in the range of normoxic cultures (Fig. 3, Fig. 6). G-CSF levels in mono-cultures of tMNC were only slightly above detection limit and could not provide this anti-apoptotic effect (Fig. 6).

The decrease of cleaved PARP observed in post-hypoxic neuronal mono-cultures at Day 3 (Fig. 3) was probably induced by rising lack of energy due to increased rate of apoptosis (Fig. 1).

Noticeably, there are application-specific differences in the regulation of cytokine secretion in co-cultures with CD133^- ^fractions. Concentrations of CCL3 and G-CSF were significantly higher in indirect co-cultures than in direct co-cultures. Possibly these enhanced concentrations are responsible for a protection from apoptosis in indirect co-cultures similar to that in direct co-cultures at Day 3. Indirect co-cultivation with tMNC did not induce enhanced cytokine levels (Fig. 6) and at the same time did not exert the same neuroprotective effect on post-hypoxic SH-SY5Y neurons. This could be explained by spatial effects: paracrine released cytokines could be more effective than action of cytokines over a longer distance.

## Conclusion

In this study we investigated human umbilical cord blood derived cell populations (tMNC, CD133^+^, and CD133^-^) according to their ability to protect post-hypoxic neuronal cells.

For different reasons, as the missing systemic effects and the disregard of brain cell interactions this *in vitro *system does only simplified reflect the action of MNC after hypoxic brain lesions *in vivo*. But taken this into account, our study delivers useful indications for the *in vivo *application of such cells:

So, since purified CD133^+ ^fractions are not superior to total HUCB-MNC in mediating neuroprotective anti-apoptotic effects, expensive and time consuming stem cell separations are not necessarily needed to yield neuroprotective cell populations. Furthermore, our study underlines the importance of MNC derived soluble factors for the mediation of neuroprotective effects visible as prevention of neuronal cells from apoptosis. Therefore, future therapeutic approaches should focus on the sufficient supply of soluble anti-apoptotic mediators, to reduce post-hypoxic brain damage.

## Methods

### Preparation of HUCB samples and isolation of CD133^+ ^cells

HUCB samples of healthy full-term neonates were obtained in accordance with ethical prescripts immediately after delivery. Samples were processed and analyzed as described previously [25]. The total MNC (tMNC) fraction gained from Ficoll density gradient (Tab. 1) was stored in the gaseous phase of liquid nitrogen. Cellular sub-fractions of tMNC were characterized using CD3-Phycoerythrin (PE, Immunotech, Hamburg, Germany), CD14-Fluorescein isothiocyanate (FITC), CD16+56-PE (both, Becton-Dickinson, Franklin Lakes, NJ, USA), CD19-Allophycocyanin (APC) and CD45-FITC, (both, Beckman Coulter, Krefeld, Germany). Prior to use tMNC were thawed and stained with carboxy fluoresceindiacetate succinimidyl ester (CFSE, Invitrogen, Karlsruhe, Germany).

CD133 positive cells (CD133^+^) were isolated from HUCB-MNC using the MACS^® ^immunomagnetic positive selection protocol (Miltenyi Biotech, Bergisch Gladbach, Germany). The flow-through fraction was collected as negative fraction (CD133^-^) depleted of CD133^+^. Final populations were analyzed using a FACSCalibur flow cytometer equipped with the CellQuest™ software (both Becton-Dickinson, Franklin Lakes, NJ, USA) and characterized by the use of the following antibodies: CD133/2-PE, Miltenyi Biotech Bergisch Gladbach, Germany), CD45-FITC and CD34-APC (both Beckman Coulter, Krefeld, Germany). Isotype-identical monoclonal antibodies served as controls. CD133^+ ^and CD133^- ^fractions contained 97.38% and 0.06% CD133 positive cells, respectively.

Status of activation of tMNC and CD133^- ^was analyzed via FACS using the antibodies CD25-PE, HLA-DR (Major Histocompatibility Complex, class II, cell surface receptor)-APC (both Becton-Dickinson, Franklin Lakes, NJ, USA), CD38-PE and CD71-FITC (both Beckman Coulter, Krefeld, Germany).

### Differentiation and hypoxic induction of SH-SY5Y cells

For differentiation 0.9 × 10^4^/cm^2 ^SH-SY5Y cells were seeded in 16-mm-diameter wells (Greiner Bio-One, Frickenhausen, Germany). After 5 days Dulbecco's Modified Eagle Medium (DMEM)/10 μM all-trans retinoic acid (Sigma-Aldrich, Steinheim, Germany)/15% fetal calf serum (PAN-Biotech, Aidenbach, Germany) media was changed into DMEM-Ham's F12/10 μM RA/5 ng/ml Brain Derived Neuronal Factor [BDNF, Immunotools Biolance GmbH, Hanover, Germany]/0.1% Human Serum Albumine [HSA, PAN Biotech GmbH, Aidenbach, Germany] for another 11 days. Media were exchanged every third day. Afterwards fully matured neuronal cells were cultured under a hypoxic atmosphere (< 1% O_2_) for 48 hours [25]. Number of viable cells remained stable between Day 16 and Day 21.

### Direct and indirect co-cultivation of post-hypoxic neuronal cells with tMNC, CD133^+ ^and CD133^- ^cell fractions

Subsequent to hypoxia, direct and indirect co-cultivation with tMNC, CD133^+ ^or CD133^- ^was carried out under normoxic conditions over a period of three days. Added tMNC, CD133^- ^(4.5 × 10^5 ^cells, both) and CD133^+ ^(4.5 × 10^3 ^cells) were dissolved in 500 μl co-culture medium (DMEM-Ham's F12, 5 ng/ml BDNF and 0.1% HSA) and added to differentiated post-hypoxic neuronal SH-SY5Y cells cultivated in adequate volume of post-hypoxic medium. Cell ratio of post-hypoxic neuronal cells to MNC was 1:15 and to CD133^+ ^1:0.15. For indirect co-cultivation tMNC or CD133^- ^were added in cell impassable cell culture inserts with a pore size of 0.4 μm (Greiner Bio-One GmbH, Frickenhausen, Germany).

Prior to co-cultivation with post-hypoxic neuronal cells, tMNC as well as CD133^+ ^and CD133^- ^were labeled with CFSE.

### Cell viability assay of neuronal cells

Within direct and indirect co-cultures and control cultures the influence of tMNC, CD133^+ ^and CD133^- ^on neuronal viability was detected via i) Propidium Iodide (PI, Invitrogen, Karlsruhe, Germany) assay for necrosis and late apoptosis and ii) annexin-V assay (Becton-Dickinson, Heidelberg, Germany) for apoptosis. The PI- and annexin-V assays were performed in cell culture plates as described previously [25]. tMNC, CD133^+ ^and CD133^- ^were distinguished from annexin-V-PE or PI positive neuronal cells by the green CFSE staining.

Cytometric Bead Array for human apoptosis (CBA, Becton Dickinson, Erembodegem, Belgium) was used to quantify the apoptosis specific parameter cleaved Poly-ADP-Ribose-Polymerase I (PARP) in lysates of post-hypoxic neuronal cells after indirect co-cultivation with tMNC and CD133^-^. For cell lyses adherent neuronal cells were rinsed with PBS and incubated on ice in the supplied buffer for 20 minutes. For analyses pooled samples obtained from three independent experiments were used.

### Cytokine profiling

For cytokine profiling supernatants of direct and indirect co-cultures were analyzed on Day 3. Supernatants of tMNC, CD133^+ ^and CD133^- ^mono-cultures and those of post-hypoxic neuronal cells were also investigated on Day 3. Cytokines were simultaneously measured using CBA for human soluble proteins (Becton Dickinson, Erembodegem, Belgium). Supernatants were screened for the following cytokines: CCL2, CCL3, CCL5, CXCL8, CXCL9 and for the growth factors basic Fibroblast Growth Factor (bFGF), Granulocyte Colony-Stimulating Factor (G-CSF), Granulocyte-Macrophage Colony-Stimulating Factor (GM-CSF) and Vascular Endothelial Growth Factor (VEGF). The detection limit was 20 pg/ml, except for CCL2 and VEGF (40 pg/ml).

### Statistical analyses of data

Except for apoptosis and necrosis rates all results have been reported as mean ± SD. Statistical differences were analyzed by Student's t-test or Mann-Whitney rank sum-test. P values of ≤ 0.05 were considered statistically significant (* p ≤ 0.05, ** p ≤ 0.01, *** p ≤ 0.001). Apoptosis and necrosis rates were logit-transformed to obtain normally distributed quantities. The effects of time, experimental setting (post-hypoxia), experimental run and the investigated well were determined univariately, and, finally multivariately using a mixed-model approach with time and experimental setting as fixed effects and well and experimental run as random effects.

Cytokine concentrations of indirect and direct co-cultures with tMNC, CD133^- ^and CD133^+ ^were compared with the sum of the concentrations obtained after post-hypoxia of neuronal cells and corresponding tMNC, CD133^+ ^and CD133^- ^mono-cultures using a bootstrapping algorithm. Therefore, we added concentrations which were resampled from e.g. the experiment post-hypoxia and the tMNC mono-culture and compared the results with concentrations obtained from e.g. the experiment of indirect co-culture with tMNC. Results were compared with Student's t-test or Mann-Whitney rank sum test. P-values reported are based on 10,000 bootstrapping simulations.

Box plots (if applicable) and univariate analyses were determined using the software package SPSS (SPSS Inc., Chicago IL, USA). Mixed Model analyses were performed using PROC MIXED of the statistical software package SAS 9.1 (SAS Institute Inc., Cary, NC, USA). Bootstrapping analysis was performed using the statistical software package "R" [31].

## Authors' contributions

DMR and SH coordinated and conducted all experimental work and wrote the manuscript. MK and TS helped to interpret the data and supported writing the manuscript. MS performed the statistical analysis. JB, WN and FE critically revised the manuscript.
